# Sexual behaviors at home and abroad: an online survey of Canadian young adult travelers

**DOI:** 10.1186/s12889-022-13383-7

**Published:** 2022-05-13

**Authors:** Emmanuelle Gareau, Karen P. Phillips

**Affiliations:** Interdisciplinary School of Health Sciences, Faculty of Health Sciences, University of Ottawa, 25 University Private, Ottawa, Ontario K1N 6N5 Canada

**Keywords:** Sexual health, Public health, Risk factors, Sexual behavior, Young adult, Sexually transmitted infections, Travel

## Abstract

**Background:**

For young adults, travel- an accessible and aspirational experience- may be accompanied by high-risk lifestyle behaviors abroad, which in turn, increases the risk of sexually transmitted and blood-borne infections (STBBI). This study aimed to examine sexual and risk behaviors of young Canadian adults both at-home and during international travel.

**Methods:**

Sexually-active Canadians, aged 18-25 years (*N* = 646) who travelled abroad in 2016, completed an online, cross-sectional survey analyzed by descriptive statistics. Outcome measures included young Canadian adults’ lifestyle risk and sexual behaviors at-home and abroad.

**Results:**

Sexual behaviors, both penetrative and non-penetrative activities, decreased significantly (*p* < 0.001; McNemar test) abroad compared to at-home. International travel elicited a statistically significant increase in alcohol consumption compared to at-home (Wilcoxon, *z* = − 11.341, *p* < 0.001). Partner type (new trip-acquired partner) abroad was associated with a greater number of travel-acquired sexual partners (Mann-Whitney, U = 4901, *p* < 0.001), inconsistent condom use during penetrative sex (U = 7215, *p* = 0.009), and sex under the influence of alcohol (Test of Two Proportions, *p* < 0.001).

**Conclusions:**

Although many young Canadian respondents practiced abstinence in their 2016 travel, for sexually-active travelers, new partner-type was related to high risk sexual behaviors. Young Canadians exhibited sexual risk behaviors both at-home and while travelling; suggesting the need for both domestic and pre-travel sexual health interventions.

## Background

Sexual health is the intersection of physical, emotional, mental and social well-being in relation to sexuality, but is also inextricably connected with reproductive health [1]. Young adults, aged 15-24 years [2], are the leading at-risk age cohort for adverse sexual health outcomes including sexually transmitted and blood-borne infections (STBBI), sexual assault and unintended pregnancy [3]. The disinhibiting effects of excessive alcohol consumption contributes to multiple, casual sex partners [4–6], made more accessible due to dating applications [7], and inconsistent/inexpert use of condoms, which in turn leads to adverse sexual health outcomes, including STBBI [8].

More than 30 different pathogens, namely bacteria, parasites and viruses transmitted via sexual contact, can cause STBBI [9]. Common asymptomatic STBBI such as chlamydia, may go untreated in the absence of regular screening [9, 10], thereby causing long-term complications such as pelvic inflammatory disease, infertility, adverse pregnancy outcomes and increased risk of acquiring secondary STBBI like HIV [9, 11, 12]. Challenges to the control and prevention of STBBI include the emergence of antimicrobial-resistant pathogenic strains [9, 10, 13], increasing rates of STBBI [10, 12, 14], and lack of surveillance programs in developing countries [13], many of which are tourist-destination regions [15].

Travel is a major factor in the global propagation of STBBI [16–19]. The past few decades have seen a democratisation in international travel [20] due to affordable airfare, low-cost backpacking/hostel tourism, voluntourism/ecotourism and study abroad trends. Travel is perceived as an aspirational goal to shape life experiences [21–23], however it also represents opportunities for escape and hedonism, including excessive alcohol consumption and risky sexual behaviors [24, 25]. The triad of travel-associated casual sex [17, 26–28], heavy alcohol use/binge drinking [19], and condom misuse culminate in increased risk of acquiring an STBBI [29, 30]. Sexual risk activity among young travelers is increased according to trip (e.g. all-inclusive/hostel settings, spring-break, single travel partner(s)) and personal factors (e.g. male gender, single status, homosexual orientation, history of multiple partners, alcohol consumption) [17, 19, 30].

Young Canadians are keen travelers [31, 32], however only a few studies have examined their sexual behaviors while travelling [27, 33–35]. Young Canadians’ motivations for travel involve personal/interpersonal intrinsic rewards [36] with vacation travel identified as a major life goal [37]. Young adults specifically identify travel among meaningful activities impacted by the COVID-19 pandemic [38], with normal travel patterns anticipated post-pandemic.

As international travel patterns are re-established post-COVID-19, this provides an excellent opportunity to prepare pre-travel interventions for all types of young adult travelers, informed by updated evidence. Assessment of the at-home lifestyle and sexual behaviors of young Canadians adds to the literature for this population and enables investigation of how these behaviors may be modified by the experience of travel. As we currently have no recent data on the sexual health of young Canadian travelers, the aim of this study is to examine young Canadian adults’ sexual and high-risk lifestyle behaviors at-home and abroad.

## Methods

### Participants

We surveyed Canadian young adults, aged 18-25 years, who had travelled internationally in 2016, about their sexual health and sexual behaviors. We recruited participants from April to October 2017 through social media, in a purposive sampling manner to ensure regional participation. We received a total of 1585 complete responses, and limited our analysis to participants with Canadian citizenship, who had travelled internationally in 2016, identified as female (including transwomen) or male (including transmen) and who had at least one sexual partner in their lifetime (*N* = 646).

### Survey

The online survey (SimpleSurvey, OutSideSoft Solutions Inc., Quebec, Canada), available in English and French, was adapted from available literature [27, 39–41]. The survey consisted of five sections: (1) Lifestyle Practices-At-Home (e.g. alcohol, drug, tobacco habits), (2) Sexual and Reproductive Health- At-Home, (3) Travel (e.g. history, travel health visits, trip characteristics (destination, organization, travel companions), lifestyle and sexual behaviors), (4) Perceptions (e.g. sexual health, travel), and (5) Demographics; generally formatted as multiple choice with some open-ended questions. Respondents were asked to report trip organization, purpose and lifestyle/sexual behaviors in the context of their most recent 2016 international trip. Participants received details about the study, the benefits and risks associated with participation and could enter a draw for a $50 CAN Amazon gift card to be compensated for their time. All survey participants provided informed consent as specified by the Research Ethics Board at the University of Ottawa (File # H02-17-14).

### Data analysis

Survey response data was extracted to ExcelTM, incomplete and missing data removed, followed by further analysis using SPSS (v. 27) (IBM Corp., Armonk, NY). Socio-demographic characteristics reported here using descriptive statistics. As men and women traditionally exhibit different sexual risk profiles, statistical analysis was limited to home/abroad and partner-type comparisons. We used McNemar’s test [42] (crossover design) with continuity correction [43] to evaluate differences in the proportion of participants’ sexual behaviors at-home and abroad. Wilcoxon signed-rank tests was used to examine participants’ alcohol consumption at-home and abroad. For trip-sexually-active participants, test of two proportions was used to evaluate effect of partner type on whether participants planned to have sex abroad (dichotomous yes/no), if participants brought condoms on trip (dichotomous yes/no), sexual behaviors (dichotomous yes/no), and frequency of sex under the influence of alcohol (dichotomous merged yes-sometimes, yes-most of the time/no). Mann-Whitney U test was used to assess the effect of partner type on frequency of condom use during sexual contact (never, rarely/sometimes, most of the time, every time; limited to trip-sexually-active participants who reported penetrative sexual activities) and number of sexual partners during travel (1, 2-5 and 6+).

## Results

### Demographics

We recognise the importance of non-binary gender expressions, however, we only included binary (male, female) participants in the analysis to meet statistical conditions. The sample for analysis therefore consisted of 646 individuals, 83.7% (*n* = 541) identifying as female (including transwomen, *n* = 2) and 16.3% (*n* = 105) identifying as male (including transmen, *n* = 3; Table 1). A majority of participants (*n* = 518, 80.2%) identified as heterosexual and Caucasian (*n* = 564, 87.3%). Most participants either lived in Ontario or Quebec, Canada (*n* = 548, 84.8%) and were post-secondary students at the time of the survey (*n* = 546, 84.5%).

**Table 1:** Demographics

	Women (***n*** = 541)	Men (***n*** = 105)	Total (***N*** = 646)
**Mean Age (years)**	21.18 (SD^a^: 2.14)	21.28 (SD: 2.06)	21.2 (SD: 2.12)
**Sexual Orientation**			
Heterosexual	438 (81.0%)	80 (76.2%)	518 (80.2%)
Other (bisexual, homosexual, pansexual, asexual or other)	103 (19.0%)	25 (23.8%)	128 (19.8%)
**Ethno-Cultural Identity** ***Respondents could choose more than one answer***			
Caucasian/white	477 (88.2%)	87 (82.9%)	564 (87.3%)
Chinese	24 (4.4%)	5 (4.8%)	29 (4.5%)
Indigenous/Aboriginal/Native	19 (3.5%)	2 (1.9%)	21 (3.3%)
South Asian	11 (2.0%)	7 (6.7%)	18 (2.8%)
Arab	13 (2.4%)	4 (3.8%)	17 (2.6%)
Southeast Asian	15 (2.8%)	1 (1.0%)	16 (2.5%)
Black	8 (1.5%)	6 (5.7%)	14 (2.2%)
Latin American	10 (1.8%)	3 (2.9%)	13 (2.0%)
Filipino	7 (1.3%)	2 (1.9%)	9 (1.4%)
West Asian	3 (0.6%)	1 (1.0%)	4 (0.6%)
Japanese	4 (0.7%)	0 (0.0%)	4 (0.6%)
Other	9 (1.7%)	1 (1.0%)	10 (1.5%)
**Province of Residence**			
Ontario	238 (44.0%)	42 (40.0%)	280 (43.3%)
Quebec	221 (40.9%)	47 (44.8%)	268 (41.5%)
British Columbia	44 (8.1%)	10 (9.5%)	54 (8.4%)
Other (Alberta, Manitoba, New Brunswick, Nova Scotia, Saskatchewan)	38 (7.0%)	6 (5.7%)	44 (6.8%)
**Highest Level of Education Completed**			
Secondary School Diploma	205 (37.9%)	36 (34.3%)	241 (37.3%)
College Diploma (including DEC^b^)	110 (20.3%)	30 (28.6%)	140 (21.7%)
University Degree (Undergraduate, Masters, PhD or medical degree)	218 (40.3%)	39 (37.1%)	257 (39.8%)
Other	8 (1.5%)	0 (0%)	8 (1.2%)
**Occupation**			
Student	459 (84.8)	87 (82.9%)	546 (84.5%)
Full or part-time work	75 (13.9%)	17 (16.2%)	92 (14.2%)
Other/Unemployed	7 (1.3%)	1 (1.0%)	8 (1.2%)

^a^SD: standard deviation; ^b^“Diplôme d’études collégiales”; pre-university diploma equivalent to Grade 12 high school + Year 1 University (Quebec, Canada)

### Sexual history

Respondents were sexually experienced, with 1-5 lifetime sexual partners (*n* = 417, 64.6%), and a sexual debut at age 15-19 years (*n* = 514, 79.6%; Table 2). In the 12 months prior to the survey, 57.0% (*n* = 368) of the sample had only one sexual partner, whereas 17.3% (*n* = 112) had multiple sexual partners within the same time period. Common contraceptive methods used at-home in Canada in the year prior to our survey included condoms (*n* = 76.4%, 494) and oral contraceptive pills (*n* = 394, 61.0%). When asked to identify the frequency of sexual encounters under the influence of alcohol in the past 2 years, participants reported such events as occurring rarely/sometimes (*n* = 491; 76.0%). Fifty participants (7.7%) reported at least one lifetime STBBI diagnosis. Among women, 5.5% (*n* = 30) had at least one pregnancy and 3.3% (*n* = 18) had at least one abortion. Lifetime history of sexual assault as of December 31, 2015 was reported by 24.9% participants (*n* = 161).

**Table 2:** Sexual history

	Women (***n*** = 541)	Men (***n*** = 105)	Total (***N*** = 646)
**Sexual debut**			
14 years old or less	56 (10.4%)	8 (7.6%)	64 (9.9%)
15-16 years old	171 (31.6%)	33 (31.4%)	204 (31.6%)
17-19 years old	264 (48.8%)	46 (43.8%)	310 (48.0%)
20 years old or older	50 (9.2%)	18 (17.1%)	68 (10.5%)
**Method(s) contraception in past year**^**a **^***Respondents could choose more than one answer***			
Condom	405 (74.9%)	89 (84.8%)	494 (76.4%)
Oral Contraceptive Pill (OCP)	350 (64.7%)	44 (41.9%)	394 (61%)
IUD^b^	64 (11.8%)	14 (13.3%)	78 (12.1%)
Hormonal methods other than OCP	28 (5.2%)	5 (4.8%)	33 (5.1%)
None	83 (15.4%)	20 (19.0%)	103 (15.9%)
Abstinence	60 (11.1%)	11 (10.5%)	71 (11.0%)
**Number of sexual partners in lifetime**			
1	141 (26.1%)	21 (20.0%)	162 (25.1%)
2-5	210 (38.8%)	45 (42.9%)	255 (39.5%)
6-10	106 (19.6%)	17 (16.2%)	123 (19.0%)
11 or more	84 (15.5%)	22 (21%)	106 (16.4%)
**Number of sexual partners in past 12 months**^**c**^			
0	19 (3.5%)	4 (3.8%)	23 (3.6%)
1	312 (57.7%)	56 (53.3%)	368 (57.0%)
2-5	172 (31.8%)	33 (31.4%)	205 (31.7%)
6-10	26 (4.8%)	7 (6.7%)	33 (5.1%)
11 or more	12 (2.2%)	5 (4.8%)	17 (2.6%)
**Number one-night stands in lifetime**			
0	253 (46.8%)	39 (37.1%)	292 (45.2%)
1-2	168 (31.1%)	31 (29.5%)	199 (30.8%)
3-5	64 (11.8%)	14 (13.3%)	78 (12.1%)
6 or more	56 (10.4%)	21 (20%)	77 (11.9%)
**Multiple sexual partners in past 12 months**^**d**^	88 (16.3%)	24 (22.9%)	112 (17.3%)
**Ever diagnosed with STBBI**^**e**^	44 (8.1%)	6 (5.7%)	50 (7.7%)
**Use dating websites or apps**	241 (44.5%)	62 (59.0%)	303 (46.9%)
**Had sexual encounters under the influence of alcohol** ***most of the time/every time*** **in past 2 years**	44 (8.1%)	7 (6.7%)	51 (7.9%)
**Sexually assaulted prior to 2016**	155 (28.7%)	6 (5.7%)	161 (24.9%)
**Ever pregnant**	30 (5.5%)		
**Ever had abortion**	18 (3.3%)		

^a^Other/write-in responses (not shown) included Plan B, pull-out method or not specified. ^b^IUD- intrauterine device. ^c^More than one sexual partner per year could include concurrent monogamous relationships (as well as polyamorous relationships), as opposed to respondents who reported having ^d^multiple sexual partners within the same time period. ^e^STBBI diagnosis in total population (*N* = 646): chlamydia (*n* = 33), genital warts/HPV (*n* = 8), gonorrhoea (*n* = 5), genital herpes (*n* = 4), scabies (*n* = 3), pubic lice (*n* = 2), chancroid (*n* = 1), HIV (*n* = 1) and syphilis (*n* = 1)

### STBBI precautionary measures

Our sample commonly employed condoms for penetrative sex (*n* = 489, 75.7%) and asking for partner(s)'s history of STBBI (*n* = 388, 60.1%; Table 3) as STBBI precautionary measures. Frequency of condom use during sexual intercourse over the past 2 years was assessed as never, rarely, sometimes, most of the time, every time. Only 21.2% (*n* = 137) of participants reported using a condom *every time* during sexual intercourse in the past 2 years, however this may be due to non-penetrative sexual practices/monogamy/alternative forms of contraception. STBBI screening at least once per year was reported by 40.1% (*n* = 259) respondents or when beginning a new relationship or with a new partner (*n* = 161, 24.9%), with 37.2% (*n* = 240) never screened.

**Table 3:** STBBI precautionary measures at-home

	Women (*n* = 541)	Men (*n* = 105)	Total (*N* = 646)
**Precautionary measure(s) taken against STBBI at-home** ***Respondents could choose more than one answer***			
Use condom for penetrative sex	409 (75.6%)	80 (76.2%)	489 (75.7%)
Use condom and/or dental dam for oral sex	2 (0.37%)	0	2 (0.31%)
Ask for partner(s)'s history of STBBI	327 (60.5%)	61 (58.1%)	388 (60.1%)
Ask for partner(s) to be screened	153 (28.3%)	19 (18.1%)	172 (26.6%)
None	42 (7.8%)	14 (13.3%)	56 (8.7%)
**Screening Frequency**^**a**^ ***Respondents could choose more than one answer***			
Never	185 (34.2%)	55 (52.4%)	240 (37.2%)
At least one per year	224 (41.4%)	35 (33.3%)	259 (40.1%)
When beginning new relationship/new partner	149 (27.5%)	12 (11.4%)	161 (24.9%)
After unprotected sex	62 (11.5%)	4 (3.8%)	66 (10.2%)
**Used condoms during sexual contact** ***every time*** **in past 2 years**	106 (19.5%)	30 (28.6%)	137 (21.2%)
**Used condoms (or dental dam) during oral sex** ***every time*** **in past 2 years**	11 (2.0%)	6 (5.7%)	17 (2.6%)

^a^Screening frequency ‘other’ write in-response not shown

### Travel characteristics

Characteristics about participants’ international trip varied greatly, with most participants vising the United States or countries in Europe for primarily tourism/leisure or to visit family. Trip duration response data was often inconsistently entered, and thus not analyzed. Travel plans were typically arranged by family members (*n* = 236, 36.5%) and/or individuals (*n* = 235, 36.4%). Few participants (16.7%, *n* = 108) reported that their most recent 2016 international trip was arranged by an organization/group/association (Table 4). Of these respondents, less than half (44.4%; *n* = 48) participants received information or recommendations about health and safety from the organization prior to travel, and only 13.0% (*n* = 14) received sexual health guidance.

**Table 4:** Travel organizations

	Women (***n*** = 541)	Men (***n*** = 105)	Total (***N*** = 646)
**Trip Arrangements**^a^**-** ***Respondents could choose more than one answer***			
Arranged by an association/organization	89 (16.5%)	19 (18.1%)	108 (16.7%)
**Among Participants Whose Trip Was Organized by an Association/Organization**			
Information received about health and safety from the association/organization	43 (48.3%)	5 (26.3%)	48 (44.4%)
Information received about sexual health from the association/organization	12 (13.5%)	2 (10.5%)	14 (13.0%)

^a^Organizations were mainly education-related (*n* = 50, 56.2% for women; *n* = 9, 47.4% for men), humanitarian/charity/volunteer related (*n* = 22, 24.7% for women; *n* = 6, 31.6%), or tourism-related (*n* = 7, 7.9% of women; *n* = 4, 21.1% of men)

### Sexual risk behaviors at-home and abroad

During their 2016 international travel, most participants were in a romantic relationship (*n* = 341, 52.8%; Table 5), did not plan to have sex (*n* = 447, 69.2%), and did not bring external condoms with them (*n* = 490, 75.9%). Among trip-sexually active respondents, 110 (40.6%) reported a new sexual partner.

**Table 5:** Sexual activity abroad

	Women			Men			All		
	Trip Abstinent (***n*** = 319)	Trip Sexually Active (***n*** = 222)	All (***n*** = 541)	Trip Abstinent (***n*** = 56)	Trip Sexually Active (***n*** = 49)	All (***n*** = 105)	Trip Abstinent (***n*** = 375)	Trip Sexually Active (***n*** = 271)	Total (***N*** = 646)
**Relationship Status During Trip**^**a**^									
Single	137 (42.9%)	65 (29.3%)	202 (37.3%)	32 (57.1%)	26 (53.1%)	58 (55.2%)	169 (54.1%)	91 (33.6%)	260 (40.2%)
In a Romantic Relationship	162 (50.8%)	142 (64.0%)	304 (56.2%)	22 (39.3%)	15 (30.6%)	37 (35.2%)	184 (49.1%)	157 (57.9%)	341 (52.8%)
In a Sexual Partnership	16 (5.0%)	9 (4.1%)	25 (4.6%)	1 (1.8%)	5 (10.2%)	6 (5.7%)	17 (4.5%)	14 (5.2%)	31 (4.8%)
Married	2 (0.6%)	4 (1.8%)	6 (1.1%)	1 (1.8%)	3 (6.1%)	4 (3.8%)	3 (0.8%)	7 (2.6%)	10 (1.5%)
**Protective, Proactive Behaviors**									
Planned to have sex abroad	8 (2.5%)	153 (68.9%)	161 (29.8%)	5 (8.9%)	33 (67.3%)	38 (36.2%)	13 (3.5%)	186 (68.6%)	199 (30.8%)
Brought condoms on trip	27 (8.5%)	87 (39.2%)	114 (21.1%)	13 (23.2%)	29 (59.2%)	42 (40.0%)	40 (10.7%)	116 (42.8%)	156 (21.4%)
**Identity of Sexual Partner(s) During Trip**^**b**^ ***(Respondent could choose more than one option)***									
Usual sexual and/or romantic partner	_	143 (64.4%)	_	_	18 (36.7%)	_	_	161 (59.4%)	_
New partner^c^		79 (35.6%)			31 (63.3%)			110 (40.6%)	

^a^ “Other” was indicated for 4 (0.7%) women in response to relationship status. Note: relationship status was not used to categorize sexual partners on trip.^b^Usual partner- usual romantic partner, usual sexual partner(s). ^c^New partner-friend(s), other traveler(s), local citizen(s), sex worker(s), other(s); Participants who indicated at least one new partner were categorized in the ‘new partner’ group. Fifteen travelers reported both usual partners as well as new partners during travel

Masturbation (*n* = 507, 78.5%), mutual masturbation (*n* = 506, 78.3%), oral sex (*n* = 599, 92.7%) and vaginal sex (*n* = 600, 92.9%; Table 6) were sexual behaviors most commonly reported by respondents while at-home in Canada. Whereas only 1.2% (*n* = 8) respondents reported no sexual behaviors at-home, abroad this significantly increased to 42.3% (*n* = 273; χ2(1) = 261.03, *p* < 0.001). We asked participants to identify their number of sexual partners during this 2016 travel, with most participants indicating abstinence (*n* = 375, 58.0%) and subsequently categorized as 'trip abstinent', despite solo-sexual activities. A small proportion of trip abstinent participants (*n* = 13, 3.5%) planned to have sex on their 2016 trip, with 61.5% (*n* = 8) of these respondents packing condoms. This is in contrast to 68.6% of trip-sexually active travelers who planned to have sex, with less than half (46.2%) of these participants bringing condoms on the trip. Alcohol consumption (4 times per week or more) increased abroad, as did alcohol abstinence (Wilcoxon, *z* = − 11.341, *p* < 0.001; Table 6).

**Table 6:** Sexual Risk Behaviors at-Home and Abroad

	Women						Men						All					
	Trip Abstinent^**a**^ (***n*** = 319)		Trip Sexually Active (***n*** = 222)		All Women (***n*** = 541)		Trip Abstinent (***n*** = 56)		Trip Sexually Active (***n*** = 49)		All Men (***n*** = 105)		Trip Abstinent (***n*** = 375)		Trip Sexually Active (***n*** = 271)		Total (***N*** = 646)	
	Home	Abroad	Home	Abroad	Home	Abroad	Home	Abroad	Home	Abroad	Home	Abroad	Home	Abroad	Home	Abroad	Home	Abroad
**Sexual Behaviors**^**b**^ ***(Respondents could choose more than one option)***																		
Masturbation	236 (74.0%)	81 (25.4%)	172 (77.5%)	69 (31.1%)	408 (75.4%)	150 (27.7%)**	53 (94.6%)	28 (50.0%)	46 (93.9%)	27 (55.1%)	99 (94.3%)	55 (52.4%)**	289 (77.1%)	109 (29.1%)	218 (80.4%)	96 (35.4%)	507 (78.5%)	205 (31.7%)**
Mutual Masturbation- (partnered)	239 (74.9%)	0 (0%)	188 (84.7%)	130 (58.6%)	427 (78.9%)	130 (24.0%)**	41 (73.2%)	0 (0%)	38 (77.6%)	25 (51.0%)	79 (75.2%)	25 (23.8%)**	280 (74.7%)	0 (0%)	226 (83.4%)	155 (57.2%)	506 (78.3%)	155 (24.0%)**
Oral Sex	294 (92.2%)	1 (0.3%)	209 (94.1%)	163 (73.4%)	503 (93.0%)	164 (30.3%)**	52 (92.9%)	1 (1.8%)	44 (89.8%)	38 (77.6%)	96 (91.4%)	39 (37.1%)**	346 (92.3%)	2 (0.5%)	253 (93.4%)	201 (74.2%)	599 (92.7%)	203 (31.4%)**
Vaginal Sex	302 (94.7%)	1 (0.3%)	211 (95.0%)	196 (88.3%)	513 (94.8%)	197 (36.4%)**	48 (85.7%)	0 (0%)	39 (79.6%)	32 (65.3%)	87 (82.9%)	32 (30.5%)**	350 (93.3%)	1 (0.3%)	250 (92.3%)	228 (84.1%)	600 (92.9%)	229 (35.4%)**
Anal Sex	49 (15.4%)	0 (0%)	42 (18.9%)	15 (6.8%)	91 (16.8%)	15 (2.8%)**	12 (21.4%)	0 (0%)	22 (44.9%)	10 (20.4%)	34 (32.4%)	10 (9.5%)**	61 (16.3%)	0 (0%)	64 (23.6%)	25 (9.2%)	125 (19.3%)	25 (3.9%)**
Sex Toys	100 (31.3%)	2 (0.6%)	90 (40.5%)	13 (5.9%)	190 (35.1%)	15 (2.8%)**	10 (17.9%)	0 (0%)	11 (22.4%)	3 (6.1%)	21 (20.0%)	3 (2.9%)**	110 (29.3%)	2 (0.5%)	101 (37.3%)	16 (5.9%)	211 (32.7%)	18 (2.8%)**
BDSM^c^	31 (9.7%)	0 (0%)	14 (6.3%)	5 (2.3%)	45 (8.3%)	5 (0.9%)**	6 (10.7%)	0 (0%)	4 (8.2%)	1 (2.0%)	10 (9.5%)	1 (1.0%)*	37 (9.9%)	0 (0%)	18 (6.6%)	6 (2.2%)	55 (8.5%)	6 (0.9%)**
N/A^d^	6 (1.9%)	236 (74.0%)	2 (0.9%)	9 (4.1%)	8 (1.5%)	245 (45.3%)**	53 (94.6%)	28 (50.0%)	46 (93.9%)	27 (55.1%)	99 (94.3%)	55 (52.4%)**	6 (1.6%)	263 (70.1%)	2 (0.7%)	10 (3.7%)	8 (1.2%)	273 (42.3%)**
**Alcohol Consumption**^**e**^																		
Everyday	1 (0.3%)	50 (15.7%)	6 (2.7%)	53 (23.9%)	7 (1.3%)	103 (19.0%)	0 (0%)	11 (19.6%)	0 (0%)	13 (26.5%)	0 (0%)	24 (22.9%)	1 (0.3%)	61 (16.3%)	6 (2.2%)	66 (24.4%)	7 (1.1%)	127 (19.7%)
4-5 Times a Week	5 (1.6%)	48 (15.0%)	10 (4.5%)	48 (21.6%)	15 (2.8%)	96 (17.7%)	3 (4.5%)	10 (17.9%)	8 (16.3%)	10 (20.4%)	11 (10.5%)	20 (19.0%)	8 (2.1%)	58 (15.5%)	18 (6.6%)	58 (21.4%)	26 (4.0%)	116 (18.0%)
2-3 Times a Week	59 (18.5%)	62 (19.4%)	55 (24.8%)	24.3%)	114 (21.1%)	116 (21.4%)	14 (25.0%)	9 (16.1%)	16 (32.7%)	15 (30.6%)	30 (28.6%)	24 (22.9%)	73 (19.5%)	71 (18.9%)	71 (26.2%)	69 (25.5%)	144 (22.3%)	140 (21.7%)
Once a Week	110 (34.5%)	41 (12.9%)	76 (34.2%)	29 (13.1%)	186 (34.4%)	70 (12.9%)	16 (28.6%)	5 (8.9%)	15 (30.6%)	2 (4.1%)	31 (29.5%)	7 (6.7%)	126 (33.6%)	46 (12.3%)	91 (33.6%)	31 (11.4%)	217 (33.6%)	77 (11.9%)
Less than Once a Week	131 (41.1%)	37 (11.6%)	73 (32.9%)	21 (9.5%)	204 (37.7%)	58 (10.7%)	19 (33.9%)	3 (5.4%)	9 (18.4%)	5 (10.2%)	28 (26.7%)	8 (7.6%)	150 (40.0%)	40 (10.7%)	82 (30.3%)	26 (9.6%)	232 (35.9%)	66 (10.2%)
Never	13 (4.1%)	81 (25.4%)	2 (0.9%)	17 (7.7%)	15 (2.8%)	98 (18.1%)	4 (7.1%)	18 (32.1%)	1 (2.0%)	4 (8.2%)	5 (4.8%)	22 (21.0%)	17 (4.5%)	99 (26.4%)	3 (1.1%)	21 (7.7%)	20 (3.1%)	120 (18.6%)

^a^All respondents were asked to identify the number of sexual partners abroad. Those who indicated 0 sexual partners were considered ‘trip abstinent’, despite solo-sexual activities and 3 responses of oral/vaginal sex. ^b^McNemar Test used to evaluate sexual behaviors at-home and abroad in women, men, and the full sample. * *p* < 0.05; ** *p* < 0.001. Sexual behaviors ‘other’ write in-response not shown. ^c^Bondage and discipline, dominance and submission, and sadism and masochism. ^d^N/A response taken as no sexual behaviors practiced. ^e^Alcohol consumption increased abroad in all participants (Wilcoxon, z = −11.341, *p* < 0.001), women (z = −10.711, *p* < 0.001) and men (z = −3.830, *p* < 0.001)

### Influence of partner-type on sexual behaviors abroad

Among the sexually-active participants abroad, 79.3% (*n* = 215; Table 7) reported only one partner, typically their usual sexual/romantic partner (*n* = 161, 59.4%; Table 5). As a small minority of partners reported both new and usual sexual partners (*n* = 15; 5.5%) during travel, we restricted our analysis to reported sexual partner type- usual romantic/sexual partner or newly acquired partner such as a friend, local citizen, travelling companion or sex worker (Table 7). Note that four women identified more than one ‘usual romantic/sexual’ partner. As these women did not categorize these partners as any of the ‘new partner’ options, they remain included in the ‘usual partner’ group. It is unclear if these are consecutive, monogamous relationships associated with lengthy trip durations or polyamorous/open relationships.

**Table 7:** Influence of Partner-Type on Sexual Behaviors Abroad

	Women [usual partner]^**a**^ ***n*** = 143	Women [new partner]^**a**^ ***n*** = 79	All Women ***n*** = 222	Men [usual partner] ***n*** = 18	Men [new partner] ***n*** = 31	All Men ***n*** = 49	All [usual partner] ***n*** = 161	All [new partner] ***N*** = 110	TOTAL ***N*** = 271
**Planned to have sex abroad**	118 (82.5%)	35 (44.3%)**	153 (68.9%)	15 (83.3%)	18 (58.1%)	33 (67.3%)	133 (82.6%)	53 (48.2%)**	186 (68.6%)
**Brought condoms on trip**	52 (36.4%)	35 (44.3%)	87 (39.2%)	8 (44.4%)	21 (67.7%)	29 (59.2%)	60 (37.3%)	56 (50.9%)*	116 (42.8%)
**Number of sexual partners during trip**									
1	139 (97.2%)	44 (55.7%)**	183 (82.4%)	18 (100.0%)	14 (45.2%)**	32 (65.3%)	157 (97.5%)	58 (52.7%)**	215 (79.3%)
2-5	3 (2.1%)^b^	32 (40.5%)	35 (15.8%)	0 (0%)	13 (41.9%)	13 (26.5%)	3 (1.9%)	45 (40.9%)	48 (17.7%)
6+	1 (0.7%)^b^	3 (3.8%)	4 (1.8%)	0 (0%)	4 (12.9%)	4 (8.2%)	1 (0.6%)	6 (5.5%)	8 (3.3%)
**Sexual Behavior(s) Abroad** ***(could choose more than one option)***^***c***^									
**Masturbation**	35 (24.5%)	34 (43.0%)*	69 (31.1%)	6 (33.3%)*	21 (67.7%)	27 (55.1%)	41 (25.5%)	55 (50.0%)**	96 (35.4%)
**Mutual Masturbation**	89 (62.2%)	41 (51.9%)	130 (58.6%)	11 (61.1%)	14 (45.2%)	25 (51.0%)	100 (62.1%)	55 (50.0%)*	155 (57.2%)
**Oral Sex**	108 (75.5%)	55 (69.6%)	163 (73.4%)	13 (72.2%)	25 (80.6%)	38 (77.6%)	121 (75.2%)	80 (72.7%)	201 (74.2%)
**Vaginal Sex**	127 (88.8%)	69 (87.3%)	196 (88.3%)	15 (83.3%)	17 (54.8%)	32 (65.3%)	142 (88.2%)	86 (78.2%)*	228 (84.1%)
**Anal Sex**	4 (2.8%)	11 (13.9%)*	15 (6.8%)	1 (5.6%)	9 (29.0%)	10 (20.4%)	5 (3.1%)	20 (18.2%)**	25 (9.2%)
**Use of Sex Toys**	10 (7.0%)	3 (3.8%)	13 (5.9%)	1 (5.6%)	2 (6.5%)	3 (6.1%)	11 (6.8%)	5 (4.5%)	16 (5.9%)
**BDSM**^**d**^	2 (1.4%)	3 (3.8%)	5 (2.3%)	1 (5.6%)	0 (0%)	1 (2.0%)	11 (6.8%)	3 (2.7%)	14 (5.2%)
**Used condoms during sexual contact** ***every time***	33 (23.1%)	28 (35.4%)*	61 (27.5%)	5 (27.8%)	9 (29.0%)	14 (28.6%)	38 (23.6%)	37 (33.6%)*	75 (27.7%)
**Had sexual encounters under the influence of alcohol**	80 (55.9%)	63 (79.7%)**	143 (64.4%)	9 (50%)	24 (77.4%)*	33 (67.3%)	89 (55.3%)	87 (79.1%)**	176 (64.9%)
**Sexually assaulted**^**e**^	2 (1.4%)	8 (10.1%)*	10 (4.5%)	1 (5.6%)	3 (9.7%)	4 (8.2%)	3 (1.9%)	11 (10%)*	14 (5.2%)
**Diagnosed with STBBI**^**f**^ **upon return**	0	5 (6.3%)	5 (2.3%)	1 (5.6%)	3 (9.7%)	4 (8.2%)	1 (0.62%)	8 (7.3%)	9 (3.3%)

^a^Usual partner = usual romantic partner, usual sexual partner(s), New partner = friend(s), other traveler(s), local citizen(s), sex worker(s), other(s); ^b^Among women who identified their usual romantic/sexual partners, 4 identified more than one partner. It is unclear whether this was due to prolonged trip duration and represents consecutive, monogamous relationships, or polyamorous/open relationships. These women did not identify these partners as belonging to any of the ‘new partner’ categories. ^c^Sexual behaviors ‘other’ write in-response not shown. ^d^Bondage and discipline, dominance and submission, and sadism and masochism^e^An additional 7 trip-abstinent women (not shown) reported sexual assault during travel.^f^STBBI included chancroid (*n* = 1), chlamydia (*n* = 4), genital herpes (*n* = 2), genital warts/HPV (*n* = 1), HIV (*n* = 1), scabies (*n* = 1), syphilis (*n* = 1); note some respondents indicated more than one type of STBBI. * *p* < 0.05; ** *p* < 0.001. Test of two proportions used to evaluate plans to have sex abroad, brought condoms on trip, frequency of sex under the influence of alcohol, sexual behaviors and sexual assault. Frequency of condom use during sex and number of sexual partners during trip were analyzed using Mann-Whitney U test, see text for details

When asked to identify all methods of contraception used on this trip, both men and women with new sexual partners primarily identified condoms (women: 53, 67.1%; men: *n* = 19, 61.3%) and no method (women: 19, 24.0%; men: *n* = 12, 38.7%), whereas travelers with their usual sexual partners commonly identified oral contraceptive pills (women: *n* = 80, 56%; men: *n* = 7, 38.9%) and condoms (women: *n* = 58, 40.6%; men: *n* = 7, 38.9%).

Trip-sexually-active women with new partners were less likely to plan to have sex abroad (Test of two proportions, χ2 = 36.353, *p* < 0.001), but had more sexual partners (Mann-Whitney U test, U = 3315.5, z = − 7.712, *p* < 0.001), used condoms more frequently (analyzed in sample reporting penetrative practices *n* = 196; U = 3287, z = − 3.041, *p* = 0.002), had sex under the influence of alcohol (χ2 = 12.619, *p* < 0.001) and were more likely to engage in anal sex (χ2 = 10.0, *p* = 0.002) compared to trip-sexually-active women with their usual partners. Similarly, men with new trip-sexual partners reported more sexual partners (Mann-Whitney U test, U = 126, z = − 3.786, *p* < 0.001), were more likely to have sex under the influence of alcohol (χ2 = 3.893, *p* = 0.048), and to have vaginal sex (χ2 = 4.081, *p* = 0.043) compared to travelers with their usual partners. More trip-sexually-active men (*n* = 31, 63.3%) reported a new sexual partner compared to women (*n* = 79, 35.6%). Only 3.3% (*n* = 9) of the participants who were sexually-active abroad were diagnosed with a STBBI post-travel, most often reported by travelers who reported new trip-associated sexual partners (*n* = 8).

Sexual assault, defined as rape, incest or other forms of sexual violence (groping, vaginal/anal penetration, unwanted touching, unwanted oral contact), was reported by 21 participants, including 7 trip-abstinent women, and 10 trip-sexually-active women and 4 men (Table 7). Sexually-active participants with new trip-acquired partners were more likely to be sexual assaulted (χ2 = 8.831, *p* = 0.003).

## Discussion

The majority of sexual health and travel studies often focus on the acquisition of new sexual partners [27, 30, 34, 35], but not on sexual behaviors or established sexual partnerships [30]. Moreover, as these studies are designed to collect behavioral data from current travelers, often there is no opportunity to also evaluate at-home behaviors. We report here that our sample of young Canadian travelers were significantly less sexually active abroad in terms of partnered activities, compared to at-home. Our sexually-active sample reported their first sexual experience between 17 and 19 years (48.0%), consistent with national estimates [44]. History of multiple partners for men was similar to national data, however slightly more women in our sample (*n* = 210, 38.8%) reported more than one sexual partner in the 12 months prior to the survey, compared to the 29.2% women, aged 20-24 years, responding to the 2015/2016 Canadian Community Health Survey [45].

### Safer sex at-home

Our findings document the participants’ breadth of sexual practices, at-home, complementing previous studies in Canada [45, 46]. Generally, our sample of young adults reported inconsistent STBBI precautions at-home, with three-quarters identifying condom use as a precautionary method, but only about one-fifth of our mostly heterosexual sample reporting condom use “every time” during intercourse in the 2 years prior to travel, much lower than reported condom use in a population of 20-24 year old Canadians (55.1%) in a national study [45]. At-home, only a few participants reported consistent use of barrier methods (condoms, dental dam) for oral sex, suggesting a gap in sexual risk reduction. Within the context of prevention and control of STBBI, and through the lens of inclusive, diverse sexualities, non-penetrative sexual activities must also be considered potential STBBI risks. Oral sex is well established to transmit both viral and bacterial STBBI, which if untreated, can cause oropharyngeal cancers [47]. At-home, young adults requested partners’ STBBI history (60.1%), however only a small minority requested partners to be tested, and only 10.2% underwent STBBI testing themselves after unprotected sex. Estimates of STBBI transmission per specific sexual act are challenging to quantify, but essentially, relative risk (RR) of HIV transmission, for example, is greatest for receivers of anal sex (RR: 100) and vaginal sex (RR: 20), as opposed to acts of sexual penetration [48]. Thus, the biological basis for STBBI transmission supports inclusive, diverse sexual health promotion that moves beyond cis-heteronormative behaviors to ensure opportunities for health referrals, information and guidance [49].

### Risky behaviors while abroad

It is well established that travel increases risk behaviors such as alcohol, casual sex, multiple sexual partners [50–52] by eliciting a sense of hedonism, adventure and escape from social control [40, 53–56]. Studies of sexual activity and travel describe wide variations in rates of sexual intercourse and new/casual sexual partners [19, 26, 27, 39–41, 52], with an estimated prevalence of travel-associated casual sex of 20.4% [30]. Single, unpartnered travelers are typically reported as having more sexual activity during travel [19, 30, 52], however in our sample, single travelers represented only 33.6% of trip-sexually-active participants (*n* = 91). Further, we determined relationship status in our sample to be unreliable in terms of partner type, as a small proportion of partnered travelers acquired new sexual partners during travel. Due in part to social desirability, monogamous relationships are still preferred by emerging young adults, however increasingly, consensual non-monogamy is viewed neutrally or with indifference among heterosexual young adults [57], and more positively by sexual minorities [58]. Both consensual and non-consensual non-monogamy increase the number of concurrent sexual partners- an established risk factor for adverse sexual health [45, 59]- thereby contributing to adverse sexual health at-home and abroad.

As relationships are more likely to be short-term and fluid in emerging young adults [58], we framed sexual risk in terms of multiple sexual partners rather than non-monogamy or infidelity. At-home, 17.3% (*n* = 112) participants reported concurrent, multiple sexual partners in the past year; a known predictor for multiple partners while travelling [19, 40, 59]. Abroad, 20.7% (*n* = 56) of trip-sexually active participants reported more than one sexual partner during their trip, lower than an earlier study wherein 39.7 to 45.7% of British backpackers identified multiple trip-sex partners [41]. Our findings are most likely due to the large proportion of women and partnered travelers in our sample.

Although men are most frequently characterized as travel-sexualized [19, 30, 40, 60], a non-trivial proportion of women in our sample also exhibited high STBBI-risk sexual behaviors. Abroad, close to half of Canadian women with new sexual partners, did not anticipate trip-associated sex or bring condoms, but reported more than one trip-acquired sexual partner. Further, almost 15% engaged in anal sex which, as discussed, is associated with higher STBBI transmission risk [48]. Anal sex is now considered common among young heterosexual women, associated with early sexual debut, high number of sexual partners, and intoxicated during intercourse [61]. As women face differential risks of acquiring STBBI [14], which then represents a significant threat to future fertility [9, 12], pre-travel sexual health promotion should include comprehensive discussions of all sexual practices.

Abroad, most participants with new trip-acquired sexual partners reported having sex under the influence of alcohol. Alcohol consumption is associated with casual sex [40], leading to increased condom use [25], albeit ineffectively and inconsistently [25, 27, 41, 51, 52, 59, 62]. Two-thirds of travelers did not consistently use condoms despite sexual encounters with new partners. As the combined effects of alcohol and a new sexual partner contribute to improper/inconsistent condom use [39, 52], the third of new-partner travelers who used condoms “every time” may still be at risk of adverse sexual health. Thus, the moderating effects of partner-type on sexual risk behaviors, previously demonstrated in non-travelling US young adults [6, 63], together with excessive alcohol consumption [25], should be considered in the design of pre-travel sexual health promotion initiatives.

Although our analysis emphasized behaviors of trip-sexually active participants, a small proportion of these travelers exhibited protective behaviors such as planning to have sex and bringing condoms, which may reflect as yet unidentified, risk-modifying characteristics in this subpopulation. However, we noted that in general, trip-abstinent participants were 13.3 times more likely to drink at least 4 or more days per week while travelling compared to at-home. Although our study did not examine binge-drinking, our findings suggest that most travelers found more occasions to consume alcohol, thus it is likely that a greater quantity of alcohol was also consumed. As discussed, alcohol consumption contributes to sexual risk behaviors and STBBI [25, 63].

In our sample, only 3.3% of participants reported a post-travel STBBI diagnosis. This may be an underestimation as many STBBI are asymptomatic and remained undetected [12]. Alternatively, as 37.2% of our sample report they never get tested for STBBI, these cases may also have been acquired pre-travel. In response to decreased STBBI testing during the COVID-19 pandemic, the United States explored home-based sample collection kits processed by public health laboratories and supported by telehealth counselling [64]. Perhaps future advances will build on the relative success of COVID-19 rapid antigen tests to enable at-home STBBI testing which can  inform sexual health decision-making.

Significant alcohol consumption also leads to compromised situational awareness and vigilance, leading to vulnerability to sexual harassment and sexual assault [65]. Sexual assault, broadly defined here, was more likely among female travelers, and those with new sexual partners. It was evident from the few write-in responses that some participants struggled to frame incidents of non-consensual, sexual touching and deviations from negotiated contraception as sexual assault. The term ‘sexualized aggression’ has been proposed for such incidents of non-consensual sexual touching without force, coercion or incapacitation, within the continuum of sexual assault [66]. Issues of consent, bodily autonomy and respect should form the bedrock of both domestic and travel-related sexual health promotion.

### Sexual health promotion

We emphasize the need for pre-travel sexual health promotion, as sexually-active young travelers may play a vector role in transmitting STBBI in their home country [8, 26, 52, 67]. Trip-organizers (e.g. education, humanitarian or ecotourism sectors) are recommended to provide sexual health pre-travel interventions including emphasis on comprehensive sexual risk behaviors, STBBI and condom use [25, 29, 59, 67, 68]. In our study, a minority of participants (16.7%) travelled as part of a formal organization, with only 13.0% of these respondents identifying organization-provided sexual health information, compared to 44.4% who received information about health and safety. This appears to be a missed opportunity by travel organizations to promote sexual health and mitigate adverse sexual health outcomes. Travel organizers cite a general lack of time and sexual health training, along with embarrassment and discomfort with the topic [69].

Pre-travel sexual health promotion can significantly improve travelers’ sexual health knowledge [56], and in turn improve sexual health [15, 27], although effectiveness of pre-travel consultation is disputed [52, 68], perhaps due to varied travel services, traveler demographics and interventions. For general travelers, there are many travel health apps [70, 71] available which could be expanded to include sexual health risk reduction and promoted on online travel sites at the point of ticket purchase. Similar online interventions which address alcohol intoxication and sexual behaviors, provide evidence-based, targeted mechanisms to improve support for young adults studying abroad [72]. Social media is yet another easily accessible resource which could be used to promote sexual health by public health agencies and the travel industry [73]. Comprehensive, evidence-based sexual health education programs at-home not only equip young adults for future sexual health decision-making domestically [3], but also in travel settings [69].

### Strengths and limitations

Using a lens of inclusive, diverse sexualities, this study provides a comprehensive portrait of the sexual behaviors of young Canadian adults- both at-home and while travelling. Typical methods of contraception and STBBI precautionary measures are also captured. Our purposive, non-random sample is not representative of the Canadian 18–25-year population, which prevents generalisability. Our sample was predominantly heterosexual, Caucasian, female and post-secondary school graduates, possibly attributed to the use of online questionnaires and recruitment methods. The cross-sectional design of our study limits establishment of causality between variables. As all measures are self-reported, this can induce a risk of social desirability and retrospective biases.

## Conclusion

To our knowledge, this is the first study assessing and characterizing the sexual health of young Canadian travelers at-home and abroad. Although our sample was less sexually-active abroad compared to at-home, alcohol consumption significantly increased and condom use decreased while travelling. Travelers with new trip-acquired sexual partners typically had sex under the influence of alcohol and did not use condoms consistently. Together with infrequent STBBI screening, the sexual health of young Canadian adults was more at-risk while travelling. It is evident that inclusive, sexual health promotion, including STBBI screening and promotion of condom use, should be a component of both domestic and travel-associated health interventions.

## Data Availability

Most of the survey data that support the findings of this study is included in manuscript tables. Some additional data for this manuscript is currently embargoed as part of a MSc thesis. It will eventually be available at the University of Ottawa online thesis portal: https://ruor.uottawa.ca/handle/10393/242.
